# Epidemiological associations between obesity, metabolism and disease risk: are body mass index and waist-hip ratio all you need?

**DOI:** 10.1038/s41366-025-01895-2

**Published:** 2025-09-19

**Authors:** Ville-Petteri Mäkinen, Siyu Zhao, Andrei Ihanus, Tuulia Tynkkynen, Mika Ala-Korpela

**Affiliations:** 1 Systems Epidemiology, Research Unit of Population Health, Faculty of Medicine, University of Oulu, Oulu, Finland; 2 Biocenter Oulu, University of Oulu, Oulu, Finland; 3 NMR Metabolomics Laboratory, School of Pharmacy, Faculty of Health Sciences, University of Eastern Finland, Kuopio, Finland

**Keywords:** Epidemiology, Obesity

## Abstract

**Background/Objectives:**

Tracking excess adiposity at population scale is essential for managing the obesity pandemic in human populations. New formulas based on weight, height, waist and hip measurements have been suggested as better alternatives to the classic body mass index and waist-hip ratio, but the lack of systematic benchmarking on how these formulas reflect adiposity, metabolic dysfunction and clinical sequelae causes confusion on how to best monitor the health of populations.

**Subjects/Methods:**

Participants from the Northern Finland Birth Cohort 1966 were included based on data availability at the 46-year visit (2511 women and 1908 men). Cross-sectional sex-adjusted Spearman correlations with clinical biomarkers and serum and urine NMR metabolomics were calculated for body mass index (BMI), waist-hip ratio (WHR), waist-height ratio (WHER), abdominal volume index, body adiposity index, body roundness index, body shape index, conicity index and impedance-based body fat. UK biobank participants were selected based on available data at initial visit (244,947 women and 205,949 men). Prevalent and incident cases of type 2 diabetes, hypertension, liver disease and heart disease were ascertained through register linkage. Prevalent cases were predicted from adiposity measures by age- and sex-adjusted logistic regression and incident cases by age- and sex-adjusted Cox regression.

**Results:**

Adiposity measures were highly collinear and exhibited low biomolecular specificity. BMI and WHR together captured almost all body shape information related to cardiometabolic diseases. For instance, the c-statistic of the BMI & WHR model for diabetes (0.8012; CI95: 0.7963, 0.8061) was near the theoretical maximum of 0.8047. Diabetes was also predicted by WHER (0.7951; CI95: 0.7903, 0.8000). Other adiposity measures showed equal or worse prediction accuracy. This pattern repeated across multiple disease diagnoses.

**Conclusions:**

We did not observe sufficient benefits from the more recent body adiposity formulas over body mass index, waist-hip or waist-height ratio to warrant their widespread application in cardiometabolic epidemiology.

## Introduction

Body mass index (BMI) and waist-hip ratio (WHR) are established practical measures of body adiposity that are easy to use and exhibit strong epidemiological associations with dysfunctional metabolism and associated clinical diagnoses such as type 2 diabetes and cardiovascular disease [1–3]. The divergence in incident disease risk between visceral and subcutaneous was already seen in earlier comparative studies of BMI and WHR (please see ref. [2] and references therein), and we and others have since published similarities and differences in how BMI and WHR reflect various aspect of ageing and metabolism [4]. Several other adiposity measures, such as body roundness [5], are now discussed as potentially better alternatives for population surveys, public health initiatives and clinical practice. These measures share the goal of accurate indirect quantification of fat and they ultimately aim to provide better diagnostic and prognostic accuracy for adverse events [6].

From a public health perspective, the information provided by adiposity formulas vis-à-vis metabolic risk assessment at middle age (before clinical diagnoses) is a key consideration since body shape is easy to survey in the general public. However, there is a lack of comprehensive analyses that compare the adiposity formulas simultaneously against other fat measurements, molecular biomarkers of metabolism, and the risk for clinical manifestations of metabolic dysfunction such as diabetes and liver disease. There is also great interest in biological subtypes of obesity based on molecular data (as the subtypes could give rise to tailored therapies [7]) but it is not clear if the new more advanced adiposity indices reflect the molecular biology of obesity better than BMI or WHR.

Direct imaging techniques of adipose tissues using various x-ray and magnetic resonance methods comprise the gold standard for quantifying an individual’s body fat mass and distribution [8]. Information on specific fat depots is biologically relevant; for example, high amount of visceral fat is more predictive of adverse cardiometabolic features and outcomes than subcutaneous fat [2, 8]. Consequently, the latest clinical consensus supports the use of direct fat quantification for diagnostic and research applications where individualized precision is a priority [9].

Indirect measures based on height, weight, hip and waist, and screening tools such as bio-impedance devices [10], provide similar fat depot information at large scale, albeit at much reduced accuracy. Notably, confounding from age differences, ethnicity, skeletal geometry and unusual height or shape of an individual prevents the precise estimation of fat and its distribution across body parts based on simple body measurements alone [11]. To mitigate the technology gap between imaging and the measurement tape, multiple adiposity formulas have been proposed that distill biomedically relevant information from the simple measures. The goal of this study was to evaluate the utility of these formulas for the epidemiology of cardiometabolic dysfunction and diseases.

## Results & discussion

### Correlations between adiposity measures

To assess the strengths and weaknesses of adiposity measures, we first investigated their mutual redundancy in the Northern Finland Birth Cohort 1966 (NFBC1966). The NFBC1966 is a longitudinal birth cohort established to study factors affecting preterm birth and consequent morbidity in the two northern-most provinces of Finland, Oulu and Lapland [12]. Data from 2012 at the age 46 years, including serum and urine metabolomics [13, 14], were available in the present study. A total of 4419 participants (2511 women and 1908 men) with complete information on height, weight, waist, hip, clinical biomarkers and metabolomics were included in statistical analyses. Body fat and visceral fat were quantified by impedance analysis [15]. In addition to BMI and WHR, we calculated the abdominal volume index (AVI [16]), body adiposity index (BAI [17]), body roundness index (BRI [18]), a body shape index (ABSI [19]), conicity index (CI [20]) and waist-height ratio (WHER) for comparative analysis.

**Fig. 1:**
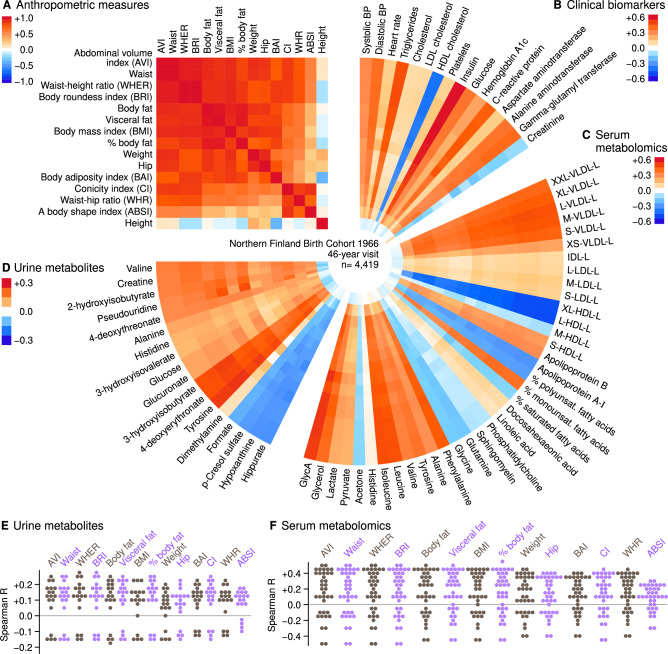
Correlation analysis of adiposity measures and selected biofluid biochemistry in the Northern Finland Birth Cohort 1966. Plots **A**–**D** are heatmap visualizations where the colour of the pixel reflects the magnitude of sex-adjusted Spearman correlation coefficient between a pair of variables. Plots **E** and **F** show the numerical distributions of coefficients from Plots **C**, **D**. BP blood pressure, GlycA glycoprotein acetyls, HDL high-density lipoprotein, IDL intermediate-density lipoprotein, LDL low-density lipoprotein, VLDL very-low-density lipoprotein. Lipoprotein subclass sizes denoted by extra small (XS) to extremely large (XXL). Total subclass lipids denoted as ‘L’.

Spearman correlation coefficients were calculated for men and women, respectively, and then meta-analysed to produce the final result. Some measures such as BRI and WHER are perfectly correlated when using the non-parametric Spearman method and there are strong correlations between all adiposity measures (Fig. 1A); hence many of the proposed measures provide limited additional information for epidemiological studies despite having more complicated formulas. Notably, BMI and WHR form one of the least redundant pairs: BMI is strongly correlated with impedance-based fat measurements, while WHR, CI and ABSI comprise a distinct collinear module.

### Correlations with serum and urine metabolomics

Next, we evaluated how well the adiposity measures captured the variation in blood-based biochemical measures that are available for epidemiological studies. We also investigated how adiposity correlated with new urine metabolomics data. Ideally, a useful measure will show meaningful correlations with biomarkers that indicate distinct disease entities (e.g. glucose for diabetes) or with causal modifiable risk factors such as low-density lipoprotein cholesterol for heart disease. When such correlations exist, the easily obtained anthropometric measurements may work as a cost-effective screening step for elevated disease risk, especially if the correlation patterns would be distinct for each adiposity measure and obesity subtype [7].

Overall, the radial stripes in Fig. 1B–D indicate a consistent correlation pattern between metabolism and adiposity measures, that is, the same sequence of positive and negative coefficients is observed for every adiposity formula. Adiposity associations were typically weaker for urine metabolites compared with clinical biomarkers or blood-based metabolic measures, please note the different colour scale in Fig. 1D. The exact magnitudes are difficult to judge based on colour alone, thus we visualized the coefficients also as dot plots in Fig. 1E, F. The correlation magnitudes were similar between BMI, body fat and WHER, while ABSI was the least correlated with metabolism.

For most of the measures, the practical differences with respect to the epidemiology of metabolic dysfunction are likely to be negligible given that there were no adiposity measures selectively associated with distinctive subsets of metabolites that would give rise to biological subtypes. This does not exclude the existence of such subtypes, on the contrary, imaging and experimental studies have revealed that metabolic specialization between fat depots and different types of adipose tissue most likely causes the divergent pattern of disease associations between visceral and subcutaneous fat, for example. The lack of specificity in this study is probably explained by the technical limitations of the available anthropometric measures as biomarkers of adiposity [11].

Clinical biomarkers and serum metabolites associated with obesity have been previously discussed and we replicated positive correlations with insulin, inflammation, triglycerides, very-low-density lipoproteins and branched chain amino acids, among others [4]. However, less is known about urine metabolites. Figure 1D includes the strongest correlations we observed and together with serum metabolites, they provide new systems insight into the population-based metabolic features of obesity.

All adiposity measures shared the same pattern of associations with urinary metabolites, and the top associations were observed for WHER and its covariate BRI. In particular, positive correlation coefficients with WHER were observed for 3-hydroxyisobutyrate (*R* = 0.250, *P* = 4.0 × 10^−64^), 4-deoxyerythronate (*R* = 0.245, *P* = 2.3 × 10^−61^) and tyrosine (*R* = 0.263, *P* = 6.1 × 10^−71^). The common theme is amino acid metabolism: 3-hydroxyisobutyrate is an intermediate of valine catabolism and implicated in insulin resistance and adipocyte function [21] and 4-deoxyerythronate is a threonine catabolism intermediate and associated with type 1 diabetes [22]. Indeed, circulating branched-chain amino acids (leucine, isoleucine and valine) and aromatic amino acids (tyrosine and phenylalanine) were increased with overweight, while glycine and glutamine were decreased (Fig. 1C), which indicates systems-wide alterations in amino acid pathways.

Negative correlation coefficients with WHER were observed for urinary formate (*R* = −0.151, *P* = 5.5 × 10^−24^), p-Cresol sulphate (*R* = −0.136, *P* = 1.3 × 10^−19^), hypoxanthine (*R* = −0.148, *P* = 4.0 × 10^−23^) and hippurate (*R* = −0.148, *P* = 5.2 × 10^−23^). Of these, p-Cresol sulphate is notable, since its precursor p-Cresol is a product from tyrosine and phenylalanine catabolism within the gut microbiome [23]. It is possible that the reduced urine concentration reflects a dietary or microbial deficiency that is part of the systemic amino acid alteration. However, further mechanistic studies are needed to infer causal relationships.

### Associations between adiposity measures and clinical diagnoses

**Fig. 2:**
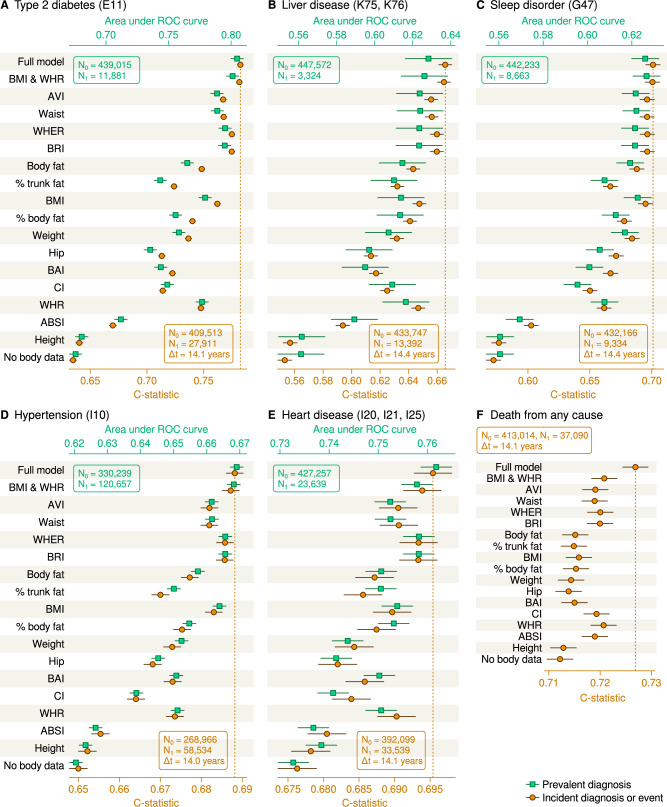
Statistical prediction of prevalent (recorded before or at the initial visit) and incident (recorded after the initial visit) diagnostic codes in the UK Biobank. The three-character codes in the plot titles refer to the International Classification of Diseases 10th Revision (Plots **A**–**E**). All models included age, sex and European ancestry status as linear confounders. The full model is the cubic polynomial surface parameterization of weight, height, waist and hip. Other models included the indicated linear term(s) and ‘No body data’ included only the confounders. Prevalent cases were predicted with logistic regression and incident cases (and mortality in Plot **F**) with Cox regression. Each plot shows the mean estimates and 95% confidence intervals for classification accuracy. N_0_ and N_1_ indicate the numbers of non-affected and affected individuals, respectively, and Δt indicates the mean follow-up time. AVI abdominal volume index, ABSI a body shape index, BAI body adiposity index, BMI body mass index, BRI body roundess index, CI conicity index, ROC receiver operator characteristic, WHR waist-hip ratio and WHER waist-height ratio.

In the second part of the study, we analysed associations between the adiposity measures and selected cardiometabolic end-points in the UK Biobank (Fig. 2, numerical results are available in Supplementary Information Tables S1 and S2). The UK Biobank is a prospective cohort study of 501,131 participants aged 37–73 years recruited between 2006 and 2010 [24]. Body fat and trunk fat were quantified by the impedance-based Tanita BC418MA body composition analyser (Tanita Europe, Amsterdam, Netherlands). Information on disease outcomes was obtained through register linkage, including Hospital Episode Statistics (HES), cancer and national death registries. In this study, 244,947 women and 205,949 men were included based on data availability.

To benchmark the information content of the adiposity measures, we created two extreme model designs that represent the best-case scenario where all the predictive information from weight, height, waist and hip combined is captured, and the null scenario where no body shape information is included. For the best-case scenario, we fitted a new model directly to the clinical event data. Theoretically, this approach will outperform any pre-defined adiposity formula (for the dataset at hand), since the model will be able to fit to the full original anthropometric dataset without extra restrictions from, say, only using the ratio of weight and height as a regressor. The above premise may not hold if the model itself is too rigid, which is possible for a simple linear model (given that many adiposity formulas are non-linear and thus less rigid). For this reason, we chose to use a cubic polynomial surface as the model design of the best-case scenario, as such a design is able to capture non-linear patterns to the same degree as the non-linear adiposity formulas. The cubic multi-variate design included height, weight, waist and hip as regressors raised to the power of 1, 2 and 3 (denoted ‘Full model’).

A linear design that included both BMI and WHR was also constructed as the state-of-the-art benchmark (denoted as ‘BMI & WHR’). Separate linear designs were constructed for each adiposity measure. Every design included age at baseline, genetic sex and European ancestry status as confounders. The ‘No body data’ design included only the confounders. For indicating prevalent disease, we used logistic regression. For predicting incident disease, we used Cox regression. Input variables were log-transformed if skewed, truncated against outlier artifacts and standardized to unit variance.

The performance of the ‘BMI & WHR’ design was on par with the full model for the diagnostic codes (Fig. 2A–E), but fell short for all-cause mortality (Fig. 2F). Similarly, WHER and its perfect co-variate BRI were almost as predictive as the full model (and the combined BMI & WHR) for prevalent and incident disease diagnoses, but did not reach the accuracy of the full model for all-cause mortality. Neither BMI nor WHR on its own exceeded the accuracy of WHER, while WHER was a better predictor of type 2 diabetes (Fig. 1A) and heart disease (Fig. 1E) than either of the two alone. These findings are important for (smaller) studies where it may be desirable to minimize the number of regressors as the relevant information can be captured by only one or two terms instead of four. In the previous study by Christakoudi and colleagues [6], ABSI predicted mortality beyond BMI classifications, however, the performance in our study was substantially lower than the full model (Fig. 2F). Towards the end of life, body mass and shape can change dramatically, which is a possible explanation why none of the arithmetic adiposity formulas could match the performance of the full non-linear prediction model for all-cause mortality [25].

### Interpretation and conclusion

We used datasets with predominantly European participants aged between 40 and 70 years that included both lean and obese phenotypes. Body height and shape depend on genetic ancestry, and we caution against extrapolating these results to other regions of the world or to other age groups. Gender is major factor that modulates associations between obesity, metabolism and disease, but we did not observe such gender effects that would substantially alter the relative performance of the adiposity measures (Supplementary Information Figs. S1 and S2). We focused on metabolism and cardiometabolic diagnoses as they are particularly relevant for population health and longevity, however, other disease conditions may not follow the same patterns. Therefore, this study is applicable to the members of the general population who are not affected by a severe illness.

Are BMI and WHR all you need for the epidemiology of obesity and cardiometabolic diseases? The short answer is yes: when combined, BMI and WHR capture the relevant population variation regarding body shape. BMI on its own was closely correlated with bioimpedance measurements of body fat, thus making it a useful proxy for body adiposity when more sophisticated techniques are not available [11]. In epidemiological studies where body shape is considered a confounder, we recommend using both BMI and WHR for model adjustment. If the focus is specifically on body fat, then using BMI alone may be justified, especially if there are socio-economic or other external reasons why height and hip circumference might be correlated with disease risk. Lastly, WHER may be a great choice to include in population surveys of metabolic dysfunction. It is very simple in concept and formula, associated with insulin resistance and type 2 diabetes, and it captures the same information as the more complicated BRI.

## Supplementary information

Supplementary material

## Data Availability

The NFBC data used in the current study are available from the cohort centre through application process for researchers who meet the criteria for access to confidential data (URL: https://www.oulu.fi/nfbc/). The UK Biobank data are available upon registration and project application (URL: https://www.ukbiobank.ac.uk/).
