# Supplementary material for: Epidemiological associations between obesity, metabolism and disease risk: are body mass index and waist-hip ratio all you need?
Source: Int J Obes (Lond). 2025 Sep 19;49(12):2555–60. doi: 10.1038/s41366-025-01895-2 (PMC12634416; doi:10.1038/s41366-025-01895-2)
Supplement: Supplementary file 1 — Supplementary material [file 41366_2025_1895_MOESM1_ESM.pdf]

Supplementary materials

Epidemiological associations between obesity, metabolism and disease risk: Are body mass index and waist-hip ratio all you need?

Ville-Petteri Mäkinen[1,2,*], Siyu Zhao[1,2], Andrei Ihanus[3], Tuulia Tynkkynen[1,2,3], Mika Ala-Korpela[1,2,3,*]

[1]Systems Epidemiology, Research Unit of Population Health, Faculty of Medicine, University of Oulu, Oulu, Finland; [2]Biocenter Oulu, University of Oulu, Oulu, Finland; [3]NMR Metabolomics Laboratory, School of Pharmacy, Faculty of Health Sciences, University of Eastern Finland, Kuopio, Finland

Contents

Table S1: Numerical prediction results for prevalent disease events
Table S2: Numerical prediction results for incident disease events
Figure S1: Prediction for prevalence and incident disease events in male participants
Figure S2: Prediction for prevalence and incident disease events in female participants

Table S1: Prediction of prevalent diagnostic codes for participants in the UK Biobank according to the International Classification of Diseases 10th Revision (ihd = I20,I21,I25; liver = K75,K76). All models included age and European ancestry status as linear confounders. The full model 'full3' is the cubic polynomial surface parameterization of weight, height, waist and hip. Other models included the indicated linear term(s) and 'empty' included only the confounders. Prevalent cases were predicted with logistic regression.

EVENT	MODEL	N.contr	N.cases	AGE	AUC	AUC.ci025	AUC.ci975
E11	empty	439015	11881	57.03	0.6758	0.6709	0.6807
G47	empty	442233	8663	57.03	0.5605	0.5544	0.5666
I10	empty	330239	120657	57.03	0.6205	0.6185	0.6225
ihd	empty	427257	23639	57.03	0.7327	0.7296	0.7358
liver	empty	447572	3324	57.03	0.5613	0.5492	0.5734
E11	full3	439015	11881	57.03	0.8047	0.7999	0.8096
G47	full3	442233	8663	57.03	0.6261	0.6200	0.6322
I10	full3	330239	120657	57.03	0.6691	0.6671	0.6710
ihd	full3	427257	23639	57.03	0.7618	0.7587	0.7649
liver	full3	447572	3324	57.03	0.6283	0.6161	0.6404
E11	bmiwhr	439015	11881	57.03	0.8012	0.7963	0.8061
G47	bmiwhr	442233	8663	57.03	0.6267	0.6205	0.6328
I10	bmiwhr	330239	120657	57.03	0.6682	0.6662	0.6701
ihd	bmiwhr	427257	23639	57.03	0.7579	0.7547	0.7610
liver	bmiwhr	447572	3324	57.03	0.6261	0.6140	0.6383
E11	BMI	439015	11881	57.03	0.7792	0.7743	0.7841
G47	BMI	442233	8663	57.03	0.6227	0.6165	0.6288
I10	BMI	330239	120657	57.03	0.6639	0.6619	0.6658
ihd	BMI	427257	23639	57.03	0.7539	0.7507	0.7570
liver	BMI	447572	3324	57.03	0.6138	0.6017	0.6259
E11	WHR	439015	11881	57.03	0.7768	0.7719	0.7817
G47	WHR	442233	8663	57.03	0.6077	0.6016	0.6138
I10	WHR	330239	120657	57.03	0.6512	0.6492	0.6532
ihd	WHR	427257	23639	57.03	0.7506	0.7475	0.7537
liver	WHR	447572	3324	57.03	0.6164	0.6042	0.6285
E11	WHER	439015	11881	57.03	0.7951	0.7903	0.8000
G47	WHER	442233	8663	57.03	0.6215	0.6154	0.6276
I10	WHER	330239	120657	57.03	0.6656	0.6637	0.6676
ihd	WHER	427257	23639	57.03	0.7583	0.7552	0.7614
liver	WHER	447572	3324	57.03	0.6234	0.6113	0.6356
E11	BAI	439015	11881	57.03	0.7438	0.7389	0.7487
G47	BAI	442233	8663	57.03	0.6009	0.5948	0.6070
I10	BAI	330239	120657	57.03	0.6508	0.6489	0.6528
ihd	BAI	427257	23639	57.03	0.7502	0.7471	0.7534
liver	BAI	447572	3324	57.03	0.5950	0.5829	0.6072
E11	BRI	439015	11881	57.03	0.7949	0.7900	0.7998
G47	BRI	442233	8663	57.03	0.6214	0.6153	0.6275
I10	BRI	330239	120657	57.03	0.6656	0.6636	0.6676
ihd	BRI	427257	23639	57.03	0.7583	0.7551	0.7614
liver	BRI	447572	3324	57.03	0.6233	0.6112	0.6355

E11	CI	439015	11881	57.03	0.7490	0.7441	0.7539
G47	CI	442233	8663	57.03	0.5955	0.5894	0.6016
I10	CI	330239	120657	57.03	0.6388	0.6368	0.6407
ihd	CI	427257	23639	57.03	0.7408	0.7377	0.7440
liver	CI	447572	3324	57.03	0.6092	0.5971	0.6214
E11	ABSI	439015	11881	57.03	0.7119	0.7070	0.7168
G47	ABSI	442233	8663	57.03	0.5694	0.5633	0.5755
I10	ABSI	330239	120657	57.03	0.6264	0.6245	0.6284
ihd	ABSI	427257	23639	57.03	0.7368	0.7337	0.7399
liver	ABSI	447572	3324	57.03	0.5893	0.5772	0.6015
E11	AVI	439015	11881	57.03	0.7887	0.7838	0.7936
G47	AVI	442233	8663	57.03	0.6218	0.6156	0.6279
I10	AVI	330239	120657	57.03	0.6615	0.6595	0.6635
ihd	AVI	427257	23639	57.03	0.7524	0.7493	0.7556
liver	AVI	447572	3324	57.03	0.6236	0.6115	0.6358
E11	WAIST	439015	11881	57.03	0.7889	0.7840	0.7938
G47	WAIST	442233	8663	57.03	0.6221	0.6160	0.6282
I10	WAIST	330239	120657	57.03	0.6616	0.6597	0.6636
ihd	WAIST	427257	23639	57.03	0.7525	0.7494	0.7556
liver	WAIST	447572	3324	57.03	0.6239	0.6118	0.6360
E11	WEIGHT	439015	11881	57.03	0.7582	0.7533	0.7631
G47	WEIGHT	442233	8663	57.03	0.6169	0.6108	0.6230
I10	WEIGHT	330239	120657	57.03	0.6524	0.6504	0.6544
ihd	WEIGHT	427257	23639	57.03	0.7438	0.7407	0.7469
liver	WEIGHT	447572	3324	57.03	0.6073	0.5952	0.6194
E11	HIP	439015	11881	57.03	0.7354	0.7306	0.7403
G47	HIP	442233	8663	57.03	0.6055	0.5994	0.6117
I10	HIP	330239	120657	57.03	0.6454	0.6434	0.6473
ihd	HIP	427257	23639	57.03	0.7414	0.7383	0.7446
liver	HIP	447572	3324	57.03	0.5970	0.5849	0.6092
E11	HEIGHT	439015	11881	57.03	0.6806	0.6757	0.6855
G47	HEIGHT	442233	8663	57.03	0.5605	0.5544	0.5667
I10	HEIGHT	330239	120657	57.03	0.6234	0.6214	0.6254
ihd	HEIGHT	427257	23639	57.03	0.7384	0.7353	0.7416
liver	HEIGHT	447572	3324	57.03	0.5617	0.5495	0.5738
E11	FAT.body	439015	11881	57.03	0.7650	0.7601	0.7699
G47	FAT.body	442233	8663	57.03	0.6193	0.6132	0.6254
I10	FAT.body	330239	120657	57.03	0.6574	0.6554	0.6593
ihd	FAT.body	427257	23639	57.03	0.7506	0.7475	0.7537
liver	FAT.body	447572	3324	57.03	0.6145	0.6024	0.6267
E11	FATPCT.body	439015	11881	57.03	0.7556	0.7507	0.7605
G47	FATPCT.body	442233	8663	57.03	0.6128	0.6067	0.6189
I10	FATPCT.body	330239	120657	57.03	0.6547	0.6528	0.6567
ihd	FATPCT.body	427257	23639	57.03	0.7532	0.7500	0.7563
liver	FATPCT.body	447572	3324	57.03	0.6133	0.6012	0.6255
E11	FATPCT.trunk	439015	11881	57.03	0.7434	0.7385	0.7483
G47	FATPCT.trunk	442233	8663	57.03	0.6077	0.6016	0.6138
I10	FATPCT.trunk	330239	120657	57.03	0.6501	0.6481	0.6521
ihd	FATPCT.trunk	427257	23639	57.03	0.7505	0.7474	0.7537
liver	FATPCT.trunk	447572	3324	57.03	0.6101	0.5980	0.6223

Table S2: Prediction of incident diagnostic codes for participants in the UK Biobank according to the International Classification of Diseases 10th Revision (ihd = I20,I21,I25; liver = K75,K76). All models included age and European ancestry status as linear confounders. The full model 'full3' is the cubic polynomial surface parameterization of weight, height, waist and hip. Other models included the indicated linear term(s) and 'empty' included only the confounders.Incident cases were predicted with Cox regression.

EVENT	MODEL	N.contr	N.cases	AGE	C	C.ci025	C.ci975
death	empty	413014	37090	57.03	0.7122	0.7097	0.7147
E11	empty	409513	27911	56.92	0.6348	0.6317	0.6380
G47	empty	432166	9334	57.01	0.5722	0.5664	0.5780
I10	empty	268966	58534	55.85	0.6500	0.6478	0.6522
ihd	empty	392099	33539	56.72	0.6763	0.6736	0.6790
liver	empty	433747	13392	57.02	0.5531	0.5483	0.5579
death	full3	413014	37090	57.03	0.7269	0.7244	0.7293
E11	full3	409513	27911	56.92	0.7824	0.7800	0.7849
G47	full3	432166	9334	57.01	0.7010	0.6953	0.7068
I10	full3	268966	58534	55.85	0.6882	0.6861	0.6902
ihd	full3	392099	33539	56.72	0.6954	0.6928	0.6980
liver	full3	433747	13392	57.02	0.6657	0.6612	0.6702
death	bmiwhr	413014	37090	57.03	0.7208	0.7183	0.7233
E11	bmiwhr	409513	27911	56.92	0.7815	0.7790	0.7840
G47	bmiwhr	432166	9334	57.01	0.7006	0.6949	0.7064
I10	bmiwhr	268966	58534	55.85	0.6871	0.6851	0.6892
ihd	bmiwhr	392099	33539	56.72	0.6939	0.6913	0.6965
liver	bmiwhr	433747	13392	57.02	0.6648	0.6603	0.6693
death	BMI	413014	37090	57.03	0.7159	0.7134	0.7184
E11	BMI	409513	27911	56.92	0.7622	0.7595	0.7648
G47	BMI	432166	9334	57.01	0.6950	0.6892	0.7008
I10	BMI	268966	58534	55.85	0.6830	0.6810	0.6851
ihd	BMI	392099	33539	56.72	0.6897	0.6870	0.6923
liver	BMI	433747	13392	57.02	0.6476	0.6430	0.6522
death	WHR	413014	37090	57.03	0.7207	0.7182	0.7232
E11	WHR	409513	27911	56.92	0.7476	0.7449	0.7503
G47	WHR	432166	9334	57.01	0.6616	0.6559	0.6673
I10	WHR	268966	58534	55.85	0.6736	0.6715	0.6756
ihd	WHR	392099	33539	56.72	0.6903	0.6876	0.6929
liver	WHR	433747	13392	57.02	0.6468	0.6422	0.6513
death	WHER	413014	37090	57.03	0.7200	0.7176	0.7225
E11	WHER	409513	27911	56.92	0.7749	0.7724	0.7775
G47	WHER	432166	9334	57.01	0.6965	0.6908	0.7022
I10	WHER	268966	58534	55.85	0.6858	0.6837	0.6878
ihd	WHER	392099	33539	56.72	0.6934	0.6907	0.6960
liver	WHER	433747	13392	57.02	0.6599	0.6553	0.6644
death	BAI	413014	37090	57.03	0.7150	0.7125	0.7175
E11	BAI	409513	27911	56.92	0.7225	0.7196	0.7253
G47	BAI	432166	9334	57.01	0.6667	0.6609	0.6726
I10	BAI	268966	58534	55.85	0.6730	0.6709	0.6751
ihd	BAI	392099	33539	56.72	0.6858	0.6831	0.6885
liver	BAI	433747	13392	57.02	0.6172	0.6125	0.6219

death	BRI	413014	37090	57.03	0.7200	0.7175	0.7224
E11	BRI	409513	27911	56.92	0.7748	0.7723	0.7774
G47	BRI	432166	9334	57.01	0.6964	0.6907	0.7021
I10	BRI	268966	58534	55.85	0.6857	0.6836	0.6878
ihd	BRI	392099	33539	56.72	0.6933	0.6907	0.6960
liver	BRI	433747	13392	57.02	0.6598	0.6553	0.6643
death	CI	413014	37090	57.03	0.7193	0.7168	0.7218
E11	CI	409513	27911	56.92	0.7139	0.7111	0.7168
G47	CI	432166	9334	57.01	0.6502	0.6446	0.6558
I10	CI	268966	58534	55.85	0.6640	0.6619	0.6662
ihd	CI	392099	33539	56.72	0.6839	0.6813	0.6866
liver	CI	433747	13392	57.02	0.6250	0.6203	0.6296
death	ABSI	413014	37090	57.03	0.7190	0.7165	0.7215
E11	ABSI	409513	27911	56.92	0.6699	0.6669	0.6729
G47	ABSI	432166	9334	57.01	0.6026	0.5970	0.6082
I10	ABSI	268966	58534	55.85	0.6554	0.6533	0.6575
ihd	ABSI	392099	33539	56.72	0.6805	0.6778	0.6832
liver	ABSI	433747	13392	57.02	0.5938	0.5891	0.5986
death	AVI	413014	37090	57.03	0.7191	0.7166	0.7215
E11	AVI	409513	27911	56.92	0.7673	0.7647	0.7698
G47	AVI	432166	9334	57.01	0.6963	0.6906	0.7020
I10	AVI	268966	58534	55.85	0.6820	0.6799	0.6840
ihd	AVI	392099	33539	56.72	0.6905	0.6879	0.6932
liver	AVI	433747	13392	57.02	0.6558	0.6513	0.6603
death	WAIST	413014	37090	57.03	0.7189	0.7164	0.7214
E11	WAIST	409513	27911	56.92	0.7677	0.7651	0.7702
G47	WAIST	432166	9334	57.01	0.6963	0.6906	0.7020
I10	WAIST	268966	58534	55.85	0.6819	0.6799	0.6840
ihd	WAIST	392099	33539	56.72	0.6906	0.6880	0.6932
liver	WAIST	433747	13392	57.02	0.6562	0.6517	0.6608
death	WEIGHT	413014	37090	57.03	0.7143	0.7118	0.7168
E11	WEIGHT	409513	27911	56.92	0.7366	0.7338	0.7394
G47	WEIGHT	432166	9334	57.01	0.6841	0.6783	0.6899
I10	WEIGHT	268966	58534	55.85	0.6729	0.6708	0.6750
ihd	WEIGHT	392099	33539	56.72	0.6843	0.6816	0.6870
liver	WEIGHT	433747	13392	57.02	0.6317	0.6270	0.6364
death	HIP	413014	37090	57.03	0.7139	0.7114	0.7164
E11	HIP	409513	27911	56.92	0.7131	0.7102	0.7160
G47	HIP	432166	9334	57.01	0.6712	0.6654	0.6771
I10	HIP	268966	58534	55.85	0.6681	0.6660	0.6702
ihd	HIP	392099	33539	56.72	0.6820	0.6793	0.6847
liver	HIP	433747	13392	57.02	0.6135	0.6088	0.6183
death	HEIGHT	413014	37090	57.03	0.7128	0.7103	0.7153
E11	HEIGHT	409513	27911	56.92	0.6404	0.6372	0.6435
G47	HEIGHT	432166	9334	57.01	0.5765	0.5707	0.5822
I10	HEIGHT	268966	58534	55.85	0.6522	0.6500	0.6544
ihd	HEIGHT	392099	33539	56.72	0.6782	0.6755	0.6809
liver	HEIGHT	433747	13392	57.02	0.5568	0.5521	0.5616

death	FAT.body	413014	37090	57.03	0.7151	0.7126	0.7176
E11	FAT.body	409513	27911	56.92	0.7484	0.7456	0.7511
G47	FAT.body	432166	9334	57.01	0.6880	0.6822	0.6938
I10	FAT.body	268966	58534	55.85	0.6772	0.6751	0.6793
ihd	FAT.body	392099	33539	56.72	0.6872	0.6845	0.6899
liver	FAT.body	433747	13392	57.02	0.6432	0.6386	0.6478
death	FATPCT.body	413014	37090	57.03	0.7153	0.7128	0.7178
E11	FATPCT.body	409513	27911	56.92	0.7401	0.7374	0.7429
G47	FATPCT.body	432166	9334	57.01	0.6778	0.6720	0.6836
I10	FATPCT.body	268966	58534	55.85	0.6753	0.6732	0.6774
ihd	FATPCT.body	392099	33539	56.72	0.6875	0.6848	0.6901
liver	FATPCT.body	433747	13392	57.02	0.6409	0.6363	0.6455
death	FATPCT.trunk	413014	37090	57.03	0.7149	0.7124	0.7174
E11	FATPCT.trunk	409513	27911	56.92	0.7240	0.7211	0.7268
G47	FATPCT.trunk	432166	9334	57.01	0.6666	0.6608	0.6724
I10	FATPCT.trunk	268966	58534	55.85	0.6700	0.6679	0.6721
ihd	FATPCT.trunk	392099	33539	56.72	0.6856	0.6829	0.6882
liver	FATPCT.trunk	433747	13392	57.02	0.6321	0.6275	0.6367

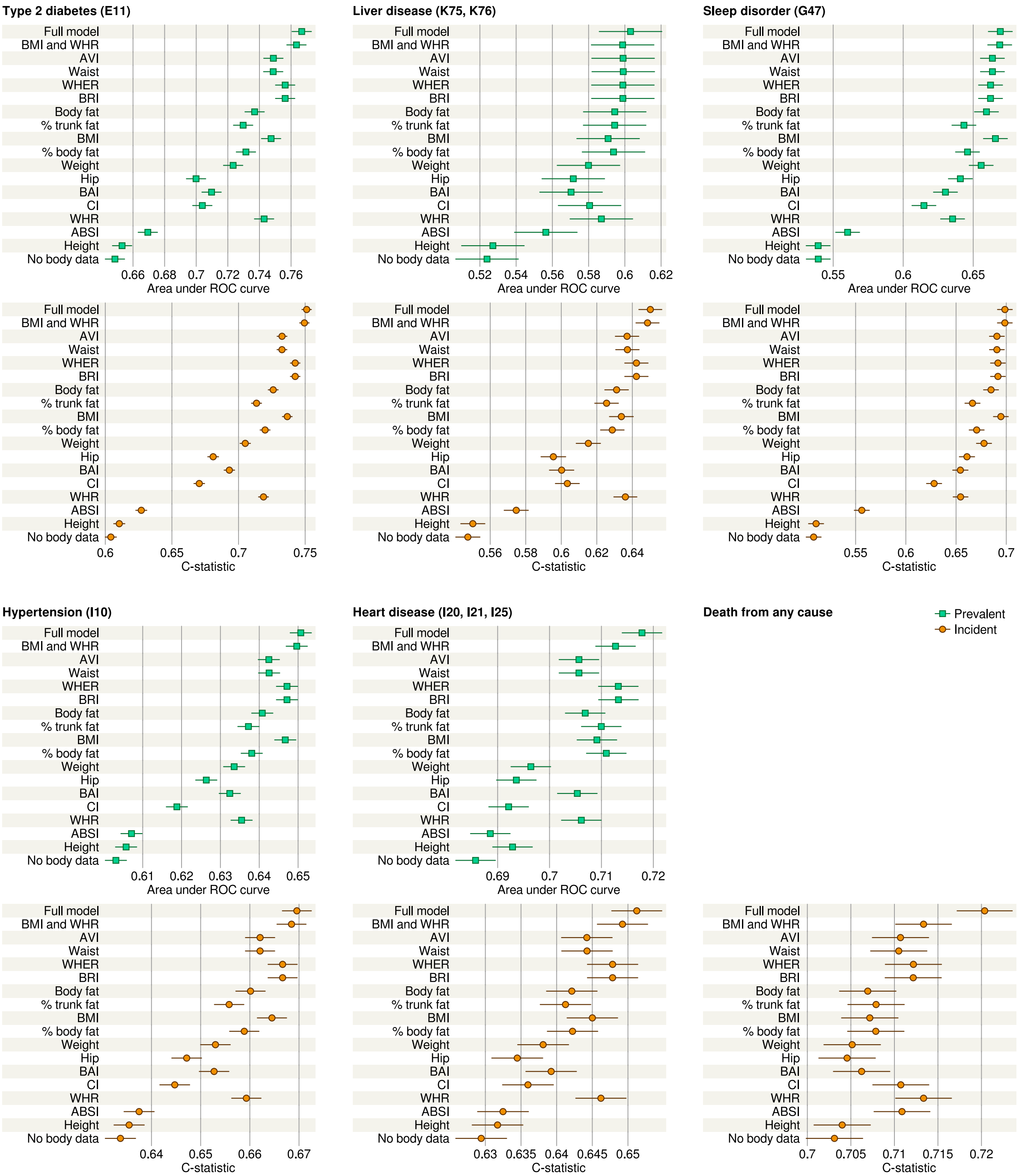

Figure S1: Prediction of diagnostic codes for male participants in the UK Biobank according to the International Classification of Diseases 10th Revision. All models included age and European ancestry status as linear confounders. The full model is the cubic polynomial surface parameterization of weight, height, waist and hip. Other models included the indicated linear term(s) and 'No body data' included only the confounders. Prevalent cases were predicted with logistic regression and incident cases with Cox regression. Abbreviations: ROC (receiver operator characteristic).

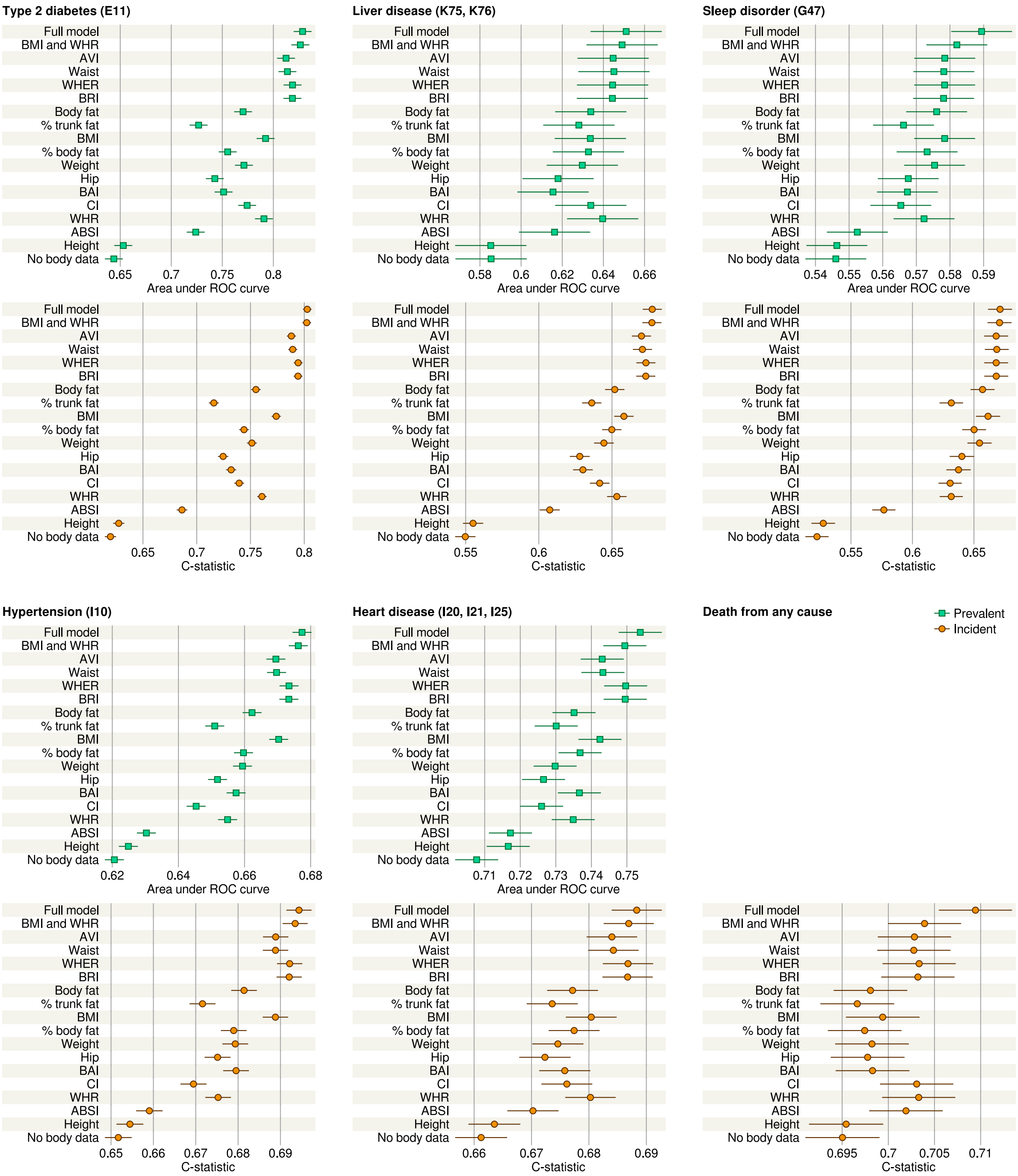

Figure S2: Prediction of diagnostic codes for female participants in the UK Biobank according to the International Classification of Diseases 10th Revision. All models included age and European ancestry status as linear confounders. The full model is the cubic polynomial surface parameterization of weight, height, waist and hip. Other models included the indicated linear term(s) and 'No body data' included only the confounders. Prevalent cases were predicted with logistic regression and incident cases with Cox regression. Abbreviations: ROC (receiver operator characteristic).
